# A Narrative Review of the Benefits of Board Games in Health

**DOI:** 10.7759/cureus.92484

**Published:** 2025-09-16

**Authors:** Eliana Alweis, Richard Alweis

**Affiliations:** 1 Biochemistry, Brown University, Providence, USA; 2 Internal Medicine, Rochester Regional Health, Rochester, USA

**Keywords:** board game, cognitive decline, mental health, patient education, preventive medicine

## Abstract

Board games and gamification - the application of game-like elements to non-game contexts - have emerged as promising strategies in healthcare for enhancing education, promoting behavioral change, and fostering social interaction. While traditionally used for entertainment, these tools are increasingly being investigated as therapeutic and diagnostic interventions across the lifespan.

This narrative review synthesizes current evidence on the use of board games in patient education, diagnosis, and treatment among pediatric, adult, elderly, and mixed-age populations. Literature was examined across preventive, diagnostic, and therapeutic domains.

The results reveal that in elderly populations, board games have demonstrated potential in maintaining cognitive health, aiding early diagnosis of cognitive impairment, and supporting individuals with mild cognitive decline, as well as increasing physical activity in nursing home residents. In adults, evidence supports benefits in preventive health education (e.g., osteoporosis, palliative care), chronic disease management, psychiatric symptom reduction, and post-surgical rehabilitation. In pediatric populations, board games have been used to improve knowledge and behaviors related to viral disease prevention, nutrition, chronic disease self-management, emotional competency, and pre-operative anxiety, with emerging evidence for attention-deficit hyperactivity disease (ADHD) symptom management. Mixed-age group interventions include risk factor modification for sexually transmitted infections, alcohol and tobacco use reduction. Across studies, board games often enhanced engagement, knowledge retention, motivation, and psychosocial well-being, though effects on long-term clinical outcomes were less consistent.

In conclusion. board games represent safe, versatile, and culturally adaptable tools for health promotion, diagnosis, and therapy. While short-term benefits in knowledge, engagement, and psychosocial outcomes are well documented, evidence for sustained clinical impact is limited. Further large-scale, methodologically rigorous studies are warranted to optimize game design for specific health objectives and evaluate long-term efficacy.

## Introduction and background

Board games have traditionally served as sources of entertainment for families, friends, and individual players. In recent years, utilization of serious games, purpose-built games focusing on improving an individual's capabilities in real-world setting, and gamification ­‑ the integration of game-like elements, such as rewards, autonomy, and mastery into non-game environments to increase engagement and motivation - have emerged as promising tools in healthcare settings [1]. These applications extend beyond enjoyment, offering avenues for education, behavioral modification, and enhanced social interaction [2].

When designing games specifically for health improvement or behavioral change, several key elements must be considered, including game mechanics, dynamics, aesthetics, and emotional engagement [2]. Studies investigating the impact of board games on health and learning employ a range of strategies: modifying everyday environments through gamification, developing purpose-built games, or adapting traditional games for new contexts. For these interventions to be effective, games must be sufficiently challenging and engaging to sustain attention without overwhelming or frustrating participants.

This narrative review summarizes the current literature on the utilization of board games in patient education, diagnosis, and treatment across the lifespan. The search strategy first involved a Pubmed search that found articles using the inquiry {("board game"[Title/Abstract]) AND (health[Title/Abstract])}. Next, all abstracts were reviewed for relevance and articles that seemed appropriate were obtained and reviewed for full text. Furthermore, both authors hand searched the bibliography to identify further articles for review. Electronic games (e-games) and video games, whether device-based or console-based, have a rich literature of their own and are beyond the scope of this review.

## Review

Section 1: Board games in the elderly population

Preventative Care

Given the high morbidity and mortality associated with cognitive decline in the elderly, multiple interventions are under study to slow progression and reduce sequelae. In this population, cognitive training interventions that target multiple cognitive abilities demonstrate greater efficacy in enhancing short- and long-term cognitive function than single-component programs focused on a specific domain [3-5]. Such interventions typically engage bilateral prefrontal and parietal regions, thereby supporting executive functions - working memory, inhibition, and cognitive flexibility - which are particularly susceptible to age-related decline, and in turn promoting cognitive and neural benefits in later life [3-5]. Board games engage these multiple brain regions, leading to the study hypothesis that the playing of board games may benefit the maintenance of cognitive health.

Both traditional and novel serious board games have shown promising results in recent studies [6]. Board games enhance logical thinking and bolster individuals’ support systems, which can help protect against cognitive decline, in addition to decreasing stress and depression levels [7]. Dartigues et al. compared individuals who did or did not play board games regularly over the course of 20 years and saw that board game players had 15% lower risk of dementia [8]. Multiple short-term studies show improvement with various games, primarily with outcomes measured as scores on a written/oral cognitive assessment, such as the Folstein Mini-Mental Status Exam (MMSE) or Montreal Cognitive Assessment (MoCA); however, longitudinal studies to confirm the results in Dartigues et al. have not been performed [7,8]. Tan hypothesized that board games were an enjoyable form of cognitive training, and individuals were more motivated to participate in the games than in traditional forms of training [9]. Differing game mechanics and activities may limit the generalizability of these studies beyond the specific cognitive domain assessed or overall global assessment [9-11]. This may also increase the difficulty in comparing an experimental game-playing group to a traditional cognitive training group beyond the specific population studied. For example, some studies have noted that modern games can significantly improve verbal fluency in older adults but do not address changes in global cognitive assessment scores [12]. Collectively, these findings suggest that while board games show promise as a tool for cognitive engagement and mental well-being, further longitudinal and domain-specific research is needed to fully understand their efficacy and broader applicability.

Diagnostic Tool

Early diagnosis of cognitive impairment provides the best opportunity for managing the disease, but these conditions are difficult to recognize at their earliest stages. Emerging evidence suggests that performance in serious game-based assessments correlates with standard cognitive test scores and may offer enhanced accuracy in the diagnosis of mild cognitive impairment and early dementia, potentially due to reduced test-related anxiety [13]. Notably, the correlations are much stronger for cognitive domains such as attention, memory, and orientation compared to other domains such as language and visuospatial skills [13]. However, there was wide heterogeneity in the types of games utilized and the specific cognitive domains tested and a recent meta-analysis concluded that this means designing specific scenarios to target areas with low correlation is important for the creation of a more holistic diagnostic tool [13]. Therefore, while board games demonstrate potential as an accessible and time-efficient tool for healthcare providers to utilize for early diagnosis, refinement is necessary to accurately examine specific cognitive domains and most effective gaming mechanics across multiple population types.

Therapeutic Care

Games are not only potentially beneficial in the early detection of cognitive decline but may also enhance function in individuals already diagnosed with mild cognitive impairment. Puzzles of varying kinds have been found to protect against cognitive decline [6,14]. Board games stimulate the midbrain and provide players with opportunities to engage in higher-order thinking, which has been shown in other models to improve cognitive function [15]. Furthermore, the environment cultivated by board games provides competition to encourage effort but does not threaten players and therefore is less likely to induce anxiety or fear [15]. Literature to date shows that cognitive training through the use of board games improved cognitive assessment scores for individuals with mild and moderate dementia, though the effect was larger for those with mild dementia [16,17]. Positive effects from board games were durable, persisting for at least three months after the trial period [16,17]. Pozzi et al. examined traditional games, ones that already exist for recreational purposes such as Chess or Go, specifically as these games are easily available, inexpensive, culturally acceptable, and shared by many within various communities [18]. Noda et al. similarly examined Ska, a traditional game of Thailand, and Shogi, a traditional two-player strategy game from Japan, have also shown positive effects on cognitive ability [18]. These studies found that while different games had slightly different effects, they all tended to improve scores on cognitive tests and memory [18,19]. Researchers also noted that the board games were easy to operate and play without prior experience, demonstrating their utility [16-17]. Additionally, to further increase ease of play, games can be facilitated by robots without disrupting the positive effects and outcomes [17].

In addition to being utilized as an adjunct in the prevention of further cognitive decline, board games can also be used to directly promote well-being and support individuals with a relatively new diagnosis [20]. Using a storytelling game, Niedderer et al. demonstrated how this activity could be harnessed to facilitate sharing and connecting to those around them while reminiscing about the past and looking toward the future [20]. Notably, some participants got upset or frustrated when they could not remember past events and they did not have high motivation to play the game outside of the context of the study [20]. In another study, Lin et al. noted that playing board games resulted in higher levels of self-efficacy and life satisfaction alongside lower levels of depression for individuals with mild cognitive impairment [17]. Studies also found that engaging in game play increases the well-being of participants by providing them with an enjoyable and social activity, especially when the difficulty level of the game is low or medium, and simply playing games with other people can reduce depression levels, especially when it is unavoidable for someone to be sedentary [10,21]. In this way, board games can be used as therapeutic tools to fight cognitive decline and increase well-being.

Nursing Home Care

Despite clear health benefits in increasing the physical activity of nursing home residents, this remains a frequently reported challenge [22,23]. Researchers found that creating an exercise game made physical activity much more enjoyable and social for residents, which increased their participation, leading to an increased number of steps and energy use per day, in addition to improved quality of life, balance, gait, and ankle strength [23]. These effects were maintained through continued participation even three months after the intervention ended [23]. However, long-term outcomes studies in these populations (e.g., reduction in hip fracture rates) are not available [23].

Section 2: Board games in the adult population

Osteoporosis

Osteoporotic fragility fractures lead to diminished quality of life, and hip fractures, in particular, have been associated with one-year mortality rates of as high as 33% [24]. With a prevalence of greater than 10% in the population aged 50 and over, education regarding the risks of the disease and preventive measures, therefore, have significant potential societal benefit from the reduction in associated morbidity and mortality of these fractures [25,26]. Tsai et al. utilized a novel board game to increase knowledge, improve attitudes, and raise engagement in preventive behaviors for osteoporosis [27]. This game helped the older adults who played convert ideas about osteoporosis from short- to long-term memory and provoked conversations between the players as they engaged in collaborative problem-solving and decision-making, the social aspect increasing the individuals’ motivation to play [27]. From a preventive standpoint, players of the game engaged in more preventative measures to protect themselves and their bones [27]. Taken as a whole, these findings highlight the potential of game-based educational tools to effectively promote osteoporosis prevention and improve long-term health outcomes among at-risk older adults.

Hip and Knee Arthroplasty

Pre- and post-operative rehabilitative exercises following total hip or knee arthroplasty are critical for optimizing recovery, minimizing long-term pain, and enhancing functional mobility and range of motion [28,29]. Early initiation of structured physiotherapy programs has been shown to accelerate post-surgical improvements in joint function and reduce the risk of complications, such as joint stiffness and muscle atrophy [28,29]. By incorporating elements of competition, including a reward-based system, and real-time performance tracking, gamification has the potential to increase motivation, leading to improved rehabilitation outcomes and greater patient satisfaction [30]. Despite its advantages in boosting engagement, studies indicate that gamified therapy does not significantly alter the amount of analgesic medication consumed postoperatively compared to standard rehabilitation programs [30]. However, the psychosocial benefits of enhanced motivation and adherence may translate into long-term improvements in mobility and reduced reliance on pain management interventions. Further randomized controlled trials are warranted to assess the broader implications of gamification in pre- and post-arthroplasty rehabilitation, particularly regarding its effects on neuromuscular adaptation, proprioception, and patient-reported functional outcomes.

Diabetes Self-Management

Diabetes self-management can be difficult for some patients to achieve due to a variety of psychosocial, educational, and cultural factors [31-33]. Studies have found that gamifying self-management improves adherence to physical exercise and nutrition standards and can help improve decision-making when working to maintain glucose levels [31]. The results are more mixed when looking at glycosylated hemoglobin levels (hereafter referred to as HbA1c), with some studies finding that gamification decreased its levels while others found no significant changes [31,32]. Furthermore, even studies which saw a decrease in HbA1c during intervention periods did not see this change sustained post intervention at follow-ups [32]. While some efficacy was shown in studies for the use of games in self-management, follow-up studies to look deeper at the changes in HbA1c levels are needed to better gauge the effectiveness of games as an intervention [32].

Psychosis

Psychotic symptoms can be difficult to diagnose and manage as they may be the result of schizophrenia, bipolar disorder, or a number of other psychiatric conditions [34]. Researchers found that simply playing novel games can improve psychosocial functioning and ease psychotic symptoms at similar levels to more traditional individualized intervention programs [35]. Khazaal et al. found improved preoccupation and conviction scores, especially for individuals with lower levels of self-reflectiveness after one session of game play [36]. This game focused specifically on cognitive restructuring and identifying alternate explanations for various situations with different degrees of psychotic and emotional loading [36]. While studies have generally found success with board game intervention, it should be noted that individuals with high levels of self-reflectiveness may require more individualized assistance [36]. It is also of note that some of the benefits from playing may result from the social interaction facilitated by the games [35]. Lau et al. found that serious games have the potential to ameliorate symptoms of depression, autism spectrum disorder, post-traumatic stress disorder, attention-deficit hyperactivity disorder, and alcohol use disorder, in addition to improving cognitive function, though there is no conclusive data from randomized controlled trials at this time [37].

End-of-Life Planning and Palliative Care

Engaging in advance care planning (ACP), as well as discussions surrounding palliative and end-of-life care, often poses significant emotional and psychological challenges for patients and their families. Conversations are commonly noted to be associated with discomfort, distress, and negative emotional responses, which can be substantial barriers to participation [38]. Providers also have difficulty engaging in these discussions with patients due to lack of training, time constraints, and a perception that patients are reluctant to engage in the conversation [38]. To address these challenges and promote meaningful dialogue, multiple studies have looked at cooperative board or card games [38-41]. These games create a space for the individual and their family to discuss experiences, desires, and needs by harnessing game pieces with images or statements to help people express themselves [39]. The game format increases motivation to engage in conversation, which, in turn, can also lead to increased engagement in ACP [39]. For example, Fernandes et al. found that among participants in a novel board game, 78% participated in at least one aspect of advanced care planning within three months of game play [39]. Additionally, games based around palliative care can help health professionals take better care of the patient and promote higher levels of communication between both groups [39]. Consequently, the incorporation of structured, game-based interventions into ACP and palliative care discussions represents a possible approach to mitigating communication barriers and enhancing collaborative decision-making among patients, families, and healthcare professionals.

Section 3: Board games in the pediatric population

Viral Disease Education

Since the COVID-19 pandemic, there has been increased interest in the use of board games to educate young children about viral diseases, including transmission, symptoms, and measures to be adopted for safety [42,43]. Board games, both in online and physical formats, can help keep students interested in their learning, which enhances knowledge acquisition [44]. Children are much more likely to be able to engage meaningfully with board games than in traditional methods of public health education, such as leaflets and posters [42,45,46]. Studies have shown that playing novel board games tailored to teach about viruses results in significantly higher knowledge than before playing the game [42,43]. While long-term outcomes remain unclear, these studies collectively indicate that public health education can be enhanced via gamification with the potential to minimize transmission of infectious diseases.

Mental Health Education

Mental health disorders are frequently associated with social stigma, which can adversely affect individuals experiencing these conditions by exacerbating psychological distress and reducing the likelihood of receiving adequate support from peers [47]. This is especially harmful since peers are often teenagers’ preferred resource for help when they are having difficulties with mental health [48-50]. Moreover, health literacy is a critical determinant of health outcomes; therefore, enhancing knowledge about mental health disorders may facilitate the adoption of appropriate health-related behaviors. Respati et al. examined the effect of a novel board game on stigma and involvement of adolescents with regard to mental health disorders [47]. This study found that playing the game led to decreased stigma and fear surrounding mental health disorders in addition to facilitating and enhancing peer roles in support systems [47].

Many serious games regarding mental health are aimed at preventing, detecting, or improving anxiety and depression [51]. Preventative games are mainly targeted at addressing emotional regulation and teaching skills, while games to increase awareness are generally meant to reduce stigma [51]. The majority of these games address prevention over therapy, though this could be due to a smaller number of individuals who benefit from a therapeutic compared to a preventative tool [51]. Martinez et al. note that games targeting depression are aimed at an adolescent audience, while games aimed at anxiety are mainly addressed to a younger childhood audience [51]. While there are many games working to elicit these effects, evidence thus far does not show statistically significant findings [52]. More studies with standardized data collection are necessary to further evaluate the utility and efficacy of serious games and gamification for the prevention and treatment of mental health disorders in children [51,52].

Nutrition Education

In the global literature, there are numerous studies investigating the efficacy of board games as interventions to promote healthy nutritional behaviors among children within their local cultural context [52-54]. Many of these studies involve games focused on education about proper nutrition to shift attitudes and perceptions in order to improve diet and exercise [53,54]. Sharps et al. focused on harnessing social norms to encourage children to eat more fruits and vegetables, as opposed to higher-calorie processed snacks [55]. The researchers found that social norms-based messaging could somewhat increase the consumption of fruits and vegetables; however, this intervention would likely be more effective if it were more specific to individual children’s contexts and home environments [55]. Using the game context allows children to enjoy learning about nutrition, though increased knowledge will not definitively change behaviors [54]. Board games can be a long-lasting educational tool that does not require continued effort from health professionals to implement, which increases their utility [56]. In contrast to some studies examining solely education, other studies combined educational measures with behavioral change encouragement through reward in the game [53,54]. These studies found that playing the novel games encouraged improved eating habits and physical activity, leading to better overall well-being [53,54]. One study also noted that participants in the game experienced fewer sick days and reported higher levels of energy [53]. These studies demonstrate the efficacy of board games to promote long-term changes in nutrition, exercise, and well-being.

Chronic Disease Management

Serious board games have been examined for their utilization in enhancing self-management of chronic disease in the pediatric population (e.g., juvenile cystic fibrosis) to serve as educational and therapeutic tools [57,58]. Self-management requires decision-making and using resources to take an active role in one’s own care [57]. Games are meant to promote engagement, encourage education, and alter behaviors to help children more effectively self-manage and improve communication with their healthcare professionals surrounding their chronic disease [57,59]. For example, Muzzolon et al. examined a novel game to assist children with atopic dermatitis to share knowledge and feelings with their healthcare provider and with peers [58]. This resulted in a better communication with those around them and improved the treatment adherence of children with atopic dermatitis [58]. Serious games were also shown to improve knowledge about asthma and relevant interventions for the parent and their child as well as demonstrating the feasibility of self-management and opening up the communication from the child to their parents and peers [60]. Beyond atopic dermatitis and asthma, serious board games can create positive change for children with cystic fibrosis or hemophilia, increasing family engagement in supporting a child with chronic illness, in addition to increasing the child’s understanding of the importance of self-care [61]. Nørlev et al. found that serious games can provide benefit for the management of type 1 diabetes in children through the incorporation of multiple different gaming mechanisms, such as goals, avatars, narrative contexts, and others [59]. More research is needed to determine which combination of mechanisms is optimal, though the mechanisms should always align with the game genre [59]. In addition to improving communication with a variety of individuals and self-management, de Jong et al. further found that play can help children cope with their chronic illness, leading to increased quality of life for both the child and their parents [62]. Stimulating play may also decrease behavioral problems in chronically ill children, though more research is necessary to determine if this trend accurately presents reality [61].

Emotional Competency Development

Numerous mental and behavioral health disorders have been associated with low levels of emotional competency (EC), particularly in children with development delays [63,64]. Due to this link, it is important to intervene early with children to help them improve their EC level. Games can facilitate learning in multiple domains in which these children struggle, for example, emotional self-regulatory and concomitant social skills and problem-solving ability [65]. They provide an opportunity to actively engage with topics in a space where children feel in control and are encouraged to persist and problem solve, thereby promoting EC. Dell’Angela et al found that games facilitate children’s implementation of EC values through play in addition to the finding that higher trait EC correlates to lower perceived effort and difficulty in playing the game, providing them a proxy through which to measure EC [65].

Serious games can encourage positive social behaviors while also decreasing negative behaviors, equip children with strategies to use during interactions, and improve communication [66,67]. They can also positively impact emotional and cognitive development by improving self-concept and emotional stability in addition to verbal intelligence and creativity [66]. Collaborative play can lead to further positive social behaviors with the cooperation necessary for these types of games [67]. Collectively, these findings suggest that serious games constitute a promising and developmentally appropriate intervention for fostering EC and associated social-cognitive skills in children, particularly those with developmental delays.

Medical Anxiety and Hospitalization Management

High levels of anxiety can cause physiological and psychological harm to children in pre-operative and hospital settings, resulting in increased pain and trauma [68,69]. Gamification of the preoperative process and playing games throughout hospitalization more generally has been investigated as a strategy to mitigate anxiety, as well as improving patient-provider communication and cooperation [68,69]. Research has shown that gamification can significantly decrease anxiety levels of children before their procedures and throughout their hospital stay [68]. Play therapy through various games can also increase child cooperation in procedures during hospitalization, additionally making the experience less negative for both acutely and chronically ill children [69].

ADHD Symptoms

Symptoms of attention deficit hyperactivity disorder (ADHD) are related to disorders of executive function abilities [70]. Games have previously been shown to support executive function in cognitive decline research, prompting researchers to examine the effects board games could have on children with ADHD [71]. Mohammed Nour ElDaou et al. demonstrated that chess training resulted in improved concentration abilities and scores on listening language tests [71]. Kim et al. found that playing Go helps enhance executive function and can be used as a non-pharmacological intervention for individuals with ADHD [72]. Furthermore, playing Go can result in changes in the EEG results when examining the prefrontal cortex, the part of the brain responsible for executive function and regulation of attention and impulses [72]. This suggests that the act of playing Go results in hyperarousal, causing the executive function and attention effects to be observed [72]. Further research could examine serious games specifically targeting ADHD to compare these effects to those of the traditional games previously tested.

Section 4: Board games in other populations

Risk Factor Modification in Mixed Age Groups: Sexually Transmitted Infections and AIDS

A significant risk factor for the acquisition of sexually transmitted infections (STIs) is insufficient knowledge regarding transmission and prevention [73-76]. Multiple studies have shown that board games can provide an enjoyable format for people to learn more about the transmission, prevention, and treatment of various STIs [73,77]. Many of these novel games contain cards that can teach facts about STIs, in addition to provoking discussion between players about this topic [73]. Wanyama et al. found that, compared to increased knowledge from a health talk, the board game format resulted in significantly higher levels of knowledge post-intervention [77]. Of note, constructing a game such that players see themselves represented in the game improves knowledge acquisition [73]. Additionally, studies on this subject typically examine short-term changes in knowledge and there is no data available yet about the long-term effects or knowledge extinction, especially relating to transmission [77]. For example, Maclachlan et al. provided youth education on AIDS through board games and demonstrated higher levels of understanding in groups that played the board game both directly after the sessions and one month after the sessions had stopped, but longer-term effects remain unknown [78].

Risk Factor Modification in Mixed Age Groups: Alcohol and Tobacco Use

Behavioral change to support the cessation of alcohol and tobacco usage is notoriously difficult both due to their addictive nature, the lack of societal stigma for continuing the behavior, and personal preferences. Those engaging in the behaviors, even to excess, are not always receptive to clinician advocacy for behavior change or receiving information about the harmful effects of the substances. Some studies have examined the use of board games on changing these attitudes and behaviors. The social connection of interactive board games can make individuals less resistant to the content of the game and even indirectly approaching their messaging can change attitudes [79]. For example, according to Czuchry et al., simply playing the game, which taught about consequences related to alcohol use, increased players’ intentions to reduce alcohol use and alter their behaviors toward alcohol [79]. Khazaal et al. have also shown that playing novel games can increase the chance of a current smoker quitting the habit [80]. In addition, games have enhanced the awareness of young adults of tactics the tobacco industry uses, which increased their negative attitudes toward these companies and products [81]. Researchers found that the board game format made the information sharing more exciting, increased investment, and created a space without guilt to converse and learn more [80].

## Conclusions

Board games can serve as therapeutic and educational interventions in a wide range of contexts for individuals of all ages. There is a large amount of research that examines these effects but much of the research is limited by small sample sizes, purpose-built games for a specific environment, low numbers of studies, and many different types of collected data. This article reviews literature covering many subjects - from nutrition in children to cognitive training in the elderly to psychosis treatment in adults. While not all research showed consistently positive findings from the use of board game or gamification interventions, there were no adverse effects reported, indicating that these measures are considered safe.

Future directions for this work include running larger scale, more standardized clinical trials with games of greater availability to obtain data of statistical significance and enhance access and reproducibility. The use of many different custom games curated for each individual study contributed to the lack of generalizable data. Additionally, these trials should expand the length of studies to interrogate long-term effects of board game play, especially in areas such as cognitive decline. Trials should more explicitly examine cultural factors or culture-specific traditional board games (e.g., work with Japanese adults and Shogi, Thai adults and Ska). Given the lack of adverse events reported in the clinical trials to date, board games appear to represent an underutilized modality in the treatment of numerous conditions.
